# Evaluation of *Megasphaera elsdenii* supplementation on rumen fermentation, production performance, carcass traits and health of ruminants: a meta-analysis

**DOI:** 10.5713/ab.22.0258

**Published:** 2023-01-10

**Authors:** Irwan Susanto, Komang G. Wiryawan, Sri Suharti, Yuli Retnani, Rika Zahera, Anuraga Jayanegara

**Affiliations:** 1 Department of Nutrition and Feed Technology, Faculty of Animal Science, IPB University, Bogor 16680, Indonesia; 2 Animal Feed and Nutrition Modelling (AFENUE) Research Group, Faculty of Animal Science, IPB University, Bogor 16680, Indonesia

**Keywords:** Acidosis, Fermentability, Health, *Megasphaera elsdenii*, Probiotics

## Abstract

**Objective:**

This study was conducted to evaluate the use of *Megasphaera elsdenii* (*M. elsdenii*) as a probiotic on rumen fermentation, production performance, carcass traits and health of ruminants by integrating data from various related studies using meta-analysis.

**Methods:**

A total of 32 studies (consisted of 136 data points) were obtained and integrated into a database. The parameters integrated were fermentation products, rumen microbes, production performance, carcass quality, animal health, blood and urine metabolites. Statistical analysis of the compiled database used a mixed model methodology. Different studies were considered random effects, while *M. elsdenii* supplementation doses were considered fixed effects. p-values and the Akaike information criterion were employed as model statistics. The model was deemed significant at p<0.05 or had a tendency to be significant when p-value between 0.05<p<0.10.

**Results:**

Supplementation with *M. elsdenii* increased (p<0.05) some proportion of fermented rumen products such as propionate, butyrate, isobutyrate, and valerate, and significantly reduced (p<0.05) lactic acid concentration, acetate proportion, total bacterial population and methane emission. Furthermore, the probiotic supplementation enhanced (p<0.05) livestock production performance, especially in the average daily gain and body condition score. Regarding the carcass quality, hot carcass weight and carcass gain were elevated (p< 0.05) due to the *M. elsdenii* supplementation. Animal health also showed improvement as indicated by the lower (p<0.05) diarrhoea and bloat incidences as well as the liver abscess. However, *M. elsdenii* supplementation had negligible effects on blood and urine metabolites of ruminants.

**Conclusion:**

Supplementation of *M. elsdenii* is capable of decreasing ruminal lactic acid concentration, enhancing rumen health, elevating some favourable rumen fermentation products, and in turn, increasing production performance of ruminants.

## INTRODUCTION

Feeding of concentrate in sufficient quantity is widely known to increase the production performance of ruminants. However, switching to excessive concentrate feeding can reduce rumen pH, resulting in metabolic disorders such as acidosis. Rumen acidosis can be chronic with detrimental effects on livestock production and health, and even result in death of livestock. Acidosis is classified into acute and sub-acute acidosis (SARA). Sub-acute acidosis happens when the pH decreases to 5.6 to 5 [1], while acute acidosis happens when the rumen pH is lower than 5 [2,3]. The decrease in pH in sub-acute acidosis is due to the accumulation of volatile fatty acids (VFA) with little detectable lactate concentration in the rumen [3]. Whereas acute acidosis is due to the fast fermentation of feed carbohydrates, which leads to the accumulation of lactic acid concentration in the rumen [1]. This condition is related to the sudden excess intake of carbohydrates into the rumen during the transition from forage to concentrate feed. This disorder is characterized by the build-up of both organic acids and lactic acid in the rumen, as lactate-utilizing microbes are less prevalent than lactic acid-producing microbes [4]. Therefore, this condition can be prevented or alleviated by increasing the population of lactate-using microorganisms or by suppressing the population of lactate-producing microorganisms.

Acidosis must be prevented because it negatively impacts animal health, including feed intake, rumen digestibility, rumen microflora, and livestock performance, and it also raises the risk of diarrhoea, laminitis, and liver abscess [5]. One of the safe and effective ways of preventing acidosis is through probiotic supplementation. Probiotic supplementation may improve feed utilization by enhancing the digestive process in the rumen. Furthermore, probiotic can restore the colonization of microorganisms lost from the digestive tract by implanting live bacteria. Bacteria as probiotics can be classified as lactic acid producing bacteria, lactic acid user bacteria, and bacteria with other functions [6]. *Megasphaera elsdenii* (*M. elsdenii*) and *Selenomonas ruminantium* (*S. ruminantium*) are the prominent lactate-consuming species in the rumen. Of the two, *S. ruminantium* has been ineffective due to catabolite repression [7] and relatively intolerant to acid [8]. In addition, *S. ruminantium* uses less lactate and only metabolizes D-lactate because it does not have the enzyme lactate racemase or L-lactate dehydrogenase to utilize L-lactate [9]. Meanwhile, *M. elsdenii* is relatively more tolerant to acid and can utilize 60% to 80% lactate in the rumen [10]. *M. elsdenii* is also able to ferment both the L and D isomers of lactate via the enzyme racemase [11] and produce propionate through the acrylate pathway [12].

A number of experiments demonstrated favourable effects of *M. elsdenii* supplementation in ruminants. For instance, supplementation of *M. elsdenii* in cattle and sheep increased propionate production in the rumen [13,14], increased feed intake by 21% and enhanced average daily weight gain [13]. The use of *M. elsdenii* of 2×10^10^ CFU in beef cattle increased feed efficiency by 19.7%, with an increase in average daily gain (ADG) of 9.7% [15]. However, several other studies also showed that *M. elsdenii* supplementation did not affect the productivity and quality of the carcass [16–18]. Apparently, variations of the supplementation doses may affect different responses and effectivity in the ruminants, and require further investigation. Our initial meta-analysis evaluation with few studies and limited number of parameters (rumen fermentation parameters only) indicated that the addition of *M. elsdenii* had a significant impact on linearly increasing rumen pH [19]. However, a more comprehensive meta-analysis study is required to integrate research findings concerning the supplementation effects of *M. elsdenii* on ruminants, and this should include all available literatures and parameters as well as the interactions with other covariates. Therefore, the purpose of this meta-analysis study was to comprehensively evaluate the effects of *M. elsdenii* administration at various doses on the characteristics of rumen fermentation, health, livestock performance and carcass quality.

## MATERIALS AND METHODS

### Development of the database

A database was compiled based on published research articles reporting the supplementation of *M. elsdenii* in ruminants. The search engines of journal collections such as Google Scholar, Web of Science, Scopus, Science Direct, Semantic Scholar, and PubMed were used to retrieve various articles on the relationship between *M. elsdenii* with rumen fermentability, productivity, and health of ruminants. Articles were included in the database if they met the following criteria: i) the articles employed *in vivo* studies in ruminants; ii) the addition of *M. elsdenii* should be the only factor in one treatment; and iii) the articles were published in English. The parameters integrated into the database were rumen pH, ammonia (NH_3_), total VFA, acetate (C_2_), propionate (C_3_), butyrate (C_4_), iso-butyrate (iso-C_4_), valerate (C_5_), iso-valerate (iso-C_5_), caproate (C_6_), ratio acetate to propionate (C_2_:C_3_), lactic acid concentration, total bacterial population, protozoa population, methane emission (CH_4_), dry matter intake (DMI), initial body weight (IBW), final body weight (FBW), ADG, body condition score (BCS), marbling, conformation, fat code, longissimus muscle area (LMA), kidney pervic and heart fat (KPH Fat), 12th rib fat, hot carcass weight, carcass gain, dressing percentage, rumen score, liver score, liver abscess, diarrhoea and bloat (DnB), pH blood, PCO_2_, blood metabolite (Na, K, Ca), blood glucose, beta-hydroxybutyric acid (BHBA), blood lactate, hematocrit, HCO_3_, excess base (BE), urine pH, urine electrolyte (Na, K, Cl), and urine electrolyte balance (EB). Outlier analysis using z-score was performed in order to ensure that the data did not have any anomaly, i.e., very different from the results of other data within a particular parameter.

The levels and strain of *M. elsdenii* supplementation were compiled in database. The final database was comprised of 32 *in vivo* studies with a total of 136 data points. The animals which received the supplementation of *M. elsdenii* were cattle and sheep with the inoculation method orally and rumen cannulated. These animals were fed primarily grains (corn grain, barley grain, wheat grain and other grains), hay, alfalfa silage, maize silage, soybean meal and concentrate. The *M. elsdenii* concentration was expressed as log 10 CFU. Measurements expressed in other units (CFU or CFU/mL) in the studies were converted to log 10 CFU using the information available in the papers. Different measuring units were used to report values for some metrics. In these situations, calculations based on the data in the articles were done to convert the values into comparable units. Methane emission was estimated using the stoichiometrical formula of VFA profiles [20]. Table 1 provides a summary of the studies included in the present meta-analysis.

### Statistical analysis

Using a mixed-model meta-analysis approach [21–23], the collected data were statistically examined. The analysis employed the PROC MIXED procedure of SAS 9.4 software (SAS Institute Inc., Cary, NC, USA) [24]. The studies were considered as random effects, while the suplementation concentrations of *M. elsdenii* were considered as the fixed effects. The continuous predictor variable consisted of the concentrations of *M. elsdenii* supplementation as follows:


$$Y_{ij}=B_{0}+B_{1}Xij+B_{2}{X_{ij}}^{2}+s_{i}+b_{i}X_{ij}+e_{ij}$$

where *Y_ij_* = dependent variable; *B_0_* = overall intercept across all studies (fixed effect); *B_1_* = linear regression coefficient of Y on X (fixed effect); *B_2_* = quadratic regression coefficient of Y on X (fixed effect); *X_ij_* = value of the continuous predictor variable (*M. elsdenii* concentration); *s_i_* = value of random effect of study i; *b_i_* = random effect of study on the regression coefficient of Y on X in study i; and *e_ij_* = the unexplained residual error. The RANDOM statement was made on the basis of different studies. The number of study replications was used to weight these models [25]. The interactions between different animals and the method of inoculation were also analyzed. To analyze the interaction, the same formula as above was employed but involving other covariates in the data, i.e., the type of livestock and the inoculation method. This model was assessed linearly as an interaction effect [26]. As for the quadratic model, when the results were not significant, the linear model was maintained. Quadratic results were selected when they were significants and had smaller AIC values. The model was deemed significant at p<0.05 or had a tendency to be significant between 0.05<p<0.10.

## RESULTS

The administration levels of *M. elsdenii* in the present study ranged between 7.00 to 13.30 log 10 CFU with an average level of 10.80±1.35 log 10 CFU. The descriptive statistical results are presented in Table 2. The influences of *M. elsdenii* supplementation on the parameter of rumen fermentation are presented in Table 3. *M. elsdenii* supplementation quadratically affected (p<0.05) ammonia and total VFA concentrations, and linearly increased (p<0.05) on rumen pH, the proportion of propionate, butyrate and isobutyrate in the rumen. The probiotic supplementation linearly decreased (p<0.001) the proportion of acetate, ratio acetate:propionate and methane. The concentration of lactic acid also decreased linearly (p<0.05) with increasing level of *M. elsdenii* supplementation. The interactions between the supplementation level with the type of animal and the method of administration were mostly insignificants; only the interaction between *M. elsdenii* level and method of adimistration was found to be significant (p<0.05) for the the concentration of lactic acid.

Parameters related to *M. elsdenii* supplementation on livestock production performance are presented in Table 4. *M. elsdenii* supplementation linearly enhanced (p<0.05) FBW, hot carcass weight, carcass gain, ADG, and BCS. However, *M. elsdenii* supplementation linearly decreased (p<0.01) DMI and 12th rib fat, and quadratically affected the kidney pervic and heart fat (p<0.05). The probiotic did not influence marbling, body conformation, fat code, LMA and dressing percentage. The interaction between supplementation level and type of animal was significant for ADG (p<0.001). Both interactions between level and type of animal as well as level and method of administration were significant (p<0.05) for the parameters of 12th rib fat and dressing percentage.

The effect of *M. elsdenii* supplementation on livestock health is presented in Table 5. Increasing level of *M. elsdenii* supplementation reduced (p<0.05) the risk of liver abscess and also the incidence of diarrhoea and bloat. Furthermore, the interactions obtained between level and different type of animal as well as different administration method were significants (p<0.05) for these parameters. The effects of *M. elsdenii* supplementation on blood and urine metabolites were not significant for most parameters (Tables 6 and 7), except that it tended to decrease (p<0.1) blood lactate and urine pH.

## DISCUSSION

### Rumen fermentation and production performance

Rumen pH is one of the most important factors to measure the fermentability of feed in the rumen. A low pH of the rumen occurs when high-concentrated feed is fed due to the rapid fermentation of starch and the accumulation of lactic acid, since the bacteria that use lactic acid have limited capacity to utilize the organic acid properly. According to the results of the meta-analysis, the administration of *M. elsdenii* can increase the rumen pH. This result is supported by a review conducted by Meissner et al [16], where *M. elsdenii* strain 41125 successfully maintained and prevented a decrease in pH during the introduction of SARA.

Furthermore, in the digestion of ruminants, feed protein that enters the rumen will be remodelled by protease enzymes produced by rumen microbes to generate NH_3_ which is the end product of feed protein degradation. According to the results of the analysis, the administration of *M. elsdenii* gave a quadratic effect on NH_3_ concentration in the rumen. The increase in ammonia at the beginning of feeding was due to the feed protein being fermented rapidly by rumen microbes. In contrast, after undergoing metabolism, the ammonia concentration decreased due to increased use of concentrates reducing the use of forage ratios which resulted in a reduced number of cellulolytic microbes that utilize NH_3_-N in the rumen [54,55]. Meanwhile, VFA, which are the main product of rumen microbial fermentation and the primary energy source for ruminants, were found to follow a quadratic response after *M. elsdenii* supplementation. An increase in VFA concentration reflects an increase in protein and soluble dietary carbohydrates in the rumen, and *vice versa* [56]. These findings demonstrate that the administration of *M. elsdenii* can modulate some products of rumen fermentation.

*M. elsdenii* is used as a probiotic because of its ability to utilize lactate in the rumen and produce propionate through the acrylate pathway [8]. According to the results of the analysis, the proportion of propionate also linearly increased following the *M. elsdenii* administration. This had been confirmed in a number of literatures as well [12–14]. In addition, the reduction of acetate proportion after *M. elsdenii* supplementation was since *M. elsdenii* at low rumen pH produce a greater proportion of propionate and butyrate than acetate [57]. Futhermore, the acetate produced is fermented again via the *β*-oxidation pathway to produce butyrate [33], which explains the increase of butyrate after the inoculation of *M. elsdenii*. With regard to such changes in the VFA profiles, it becomes clear that *M. elsdenii* is able to lower methane production since propionate formation utilizes hydrogen which is a primary substrate for methanogenesis [58].

Regarding the lower concentration of lactic acid after *M. elsdenii* supplementation, the bacterial species is able to metabolize 60% to 80% of lactic acid in the rumen [6], making it as a suitable probiotic candidate to help in mitigating lactic acidosis. Various *in vitro* and *in vivo* experiments have been conducted and generally they demonstrated that *M. elsdenii* was able to prevent the accumulation of lactic acid during the transition to high-grain diets [36,51,59–62]. The interaction between *M. elsdenii* supplementation level and different inoculation method was significant, indicating that there is an effect of the *M. elsdenii* inoculation method on the concentration of lactic acid. *M. elsdenii* is an obligate anaerobic bacterium, so it must be administered orally or via a rumen cannula to avoid oxygen exposure [47]. Several studies with oral inoculation showed more efficient results [15,47]. Oral inoculation is easier with a large number of livestock such as in feedlots, thereby reducing maintenance costs. However, fresh oral products are limited to temporary storage, since they have 14 days shelf life, and are challenging to administer. Livestock must be brought to a chute to receive the probiotic. Research related to oral administration by processing using rehydrated lyophilized has proven to maintain the viability of *M. elsdenii* cultures [18].

Supplementation of *M. elsdenii* also quadratically influences the bacterial population in the rumen. Initially, the bacterial population increased until a certain optimal level of *M. elsdenii* supplementation and then decreased afterwards. Still, after using a large amount of concentrated feed, which causes the fermentation to take place rapidly, the pH decreases due to the increased concentration of lactic acid, which triggers an overly acidic pH condition and kills some bacteria intolerant of acidic conditions. However, several studies have shown that the microbial population of *S. bovis* and *M. elsdenii* increase with the increasing level of concentrate use in the diet [1,13,45]. The interactions between *M. elsdenii* administration and different types of animals as well as inoculation methods tended to affect the bacterial population. In the present meta-analysis, the protozoa population was not affected by *M. elsdenii* administration. In another study, protozoa population increased in response to *M. elsdenii* inoculation and this was beneficial for rumen ecology since protozoa competed with *S. bovis* for substrate in the rumen, therefore minimizing the risk of sub-acute acidosis [36]. According to Wiryawan and Brooker [60], protozoa in the rumen serve as a stabilizing factor for fermentation by consuming bacteria which slows fermentation, particularly in animals fed high-grain diets.

It was revealed in the present meta-analysis that *M. elsdenii* supplementation reduced DMI. Likewise, administration of *M. elsdenii* decreased dry matter consumption in heifers [47]. Furthermore, Miller et al [17] explained that the decrease in feed consumption after being given Lacticpro, which is a culture of *M. elsdenii* strain NCIMB 41125 (Lactipro Advance) in steers did not affect livestock growth, and this difference in feed intake only occurred in the first 18 days. In addition, cattle supplemented with *M. elsdenii* consumed less DMI resulting in a 19.7% increase in feed efficiency [15]. Moreover, the effect of increasing propionate production in the rumen after being given *M. elsdenii* also can reduce DMI [14]. This statement is supported by Allen et al [63], who reviewed several studies in which increased propionate in ruminants altered the size and frequency of meals by increasing satiety, thus decrease the DMI. In fact, the decrease in DMI due to *M. elsdenii* supplementation had no negative effect on livestock performance but, on the contrary, it enhanced FBW, ADG, and BCS. These results are also in line with DeClerck et al [15], in which cows given *M. elsdenii* had higher ADG as compared to the control treatment, which correlated with an increase in BCS. In addition, steers that received *M. elsdenii* had a 5.6% better ADG at 3 to 5 weeks of maintenance [40].

### Carcass quality

*M. elsdenii* supplementation had negligible effects on marbling, conformation, fat code, and LMA. In accordance with the findings of multiple studies, administration of *M. elsdenii* had no effects on LMA [15], marbling scores [41], conformational scores and fat code [51]. However, several studies have shown that there is an increase in the marbling value due to increased lipogenesis [41], as a result of increased rumen fermentation products which are precursors of fat formation. The increase in fermented products as fat precursors actually had an effect on kidney pervic and heart fat which decreased before increasing quadraticly. However, this increase was not accompanied by 12th rib fat which decreased linearly. The decrease of rib fat was due to the interaction effect between different livestock types and inoculation methods. This result contradicts several previous studies, were all stated that *M. elsdenii* inoculation had no influence on carcass quality [39,41,42].

This study also found that *M. elsdenii* supplementation increased hot carcass weight and carcass gain. This supports the results on the productivity parameters, i.e., ADG and FBW, which increased due to the probiotic administration. This was in line with DeClerck et al [15] where *M. elsdenii* inoculation increased carcass gain by 0.20 kg as compared to control. Cattle fed *M. elsdenii* had an increase in carcass daily gain and hot carcass weight with a shorter adaptation time and oral inoculation [43]. Furthermore, Meisner et al [16] conducted a review regarding the use of *M. elsdenii* in South Africa, and it was revealed that cattle supplemented with *M. elsdenii* at 1×10^10^ CFU had a 2.2% increase in carcass ADG. An increase in ADG and the use of concentrate feed was found to be associated with dressing percentage parameters, where *M. elsdenii* supplementation tended to have a linear effect on increasing dressing percentage. These results certainly strengthen the use of *M. elsdenii* probiotics, which are not only able to utilize lactate in the rumen but also increase productivity and several parameters of carcass quality.

### Livestock health

Probiotics have the ability to maintain the balance and activity of the gastrointestinal microbiota, making them beneficial for the host animal [64]. In ruminants, rumen health is an important factor that must be taken into consideration. *M. elsdenii*, which is capable of utilizing lactic acid in the rumen, is believed to be responsible for rumen health maintenace. In the present study, although *M. elsdenii* administration did not significantly affect rumen score and liver score, it reduced the risk of livestock experiencing liver abscess and the incidence of diarrhoea and bloat. This reduced risk of liver abscess reflects a mutually beneficial rumen fermentation between *M. elsdenii* and rumen microbes [51]. Furthermore, Miller et al [17] stated that livestock given commercial probiotic *M. elsdenii* Lacticpro experienced a decrease in the incidence and severity of liver abscess, which indicated that *M. elsdenii* was effective in preventing acidosis in cattle. Meanwhile, the number of cattle that experienced diarrhoea and bloat were lower following the *M. elsdenii* supplementation. Administration of *M. elsdenii* strain NCIMB 41125 was able to reduce morbidity or livestock experiencing health problems [40].

The last is related to blood metabolites and urine electrolytes. Based on the results on the blood parameters, the results were not significantly different (p>0.1) in pH and most blood metabolites parameters, apart from the fact that the blood lactate concentration tended to decrease linearly (p = 0.054) as the level of *M. elsdenii* increased. Blood pH rarely fluctuates under normal conditions, this is due to the acid-base conditions of the blood that are saturated with HCO_3_ [36]. HCO_3_ levels indicate the importance of the kidneys in maintaining the acid-base balance of the blood [65]. Plasma lactate concentrations were lower and tended to decrease more, with wheat-based concentrate feed compared to corn-based feed given the same level of *M. elsdenii* probiotic inoculation [36]. The decrease in blood lactate is also related to the activation response of the buffer mechanism to blood acidity after being given a high-concentrated diet. These results also prove that *M. elsdenii* supplementation can minimize the risk of metabolic acidosis due to excessive concentrate feeding.

Furthermore, the urine electrolyte variables Na, K, Cl, and BE were not significantly different (p>0.1). Urinary electrolytes are associated with activation of the buffering mechanism against blood acidity by inoculation of *M. elsdenii* [36]. This is related to the value of the dietary cation-anion difference (DCAD), where increasing the proportion of concentrate facilitates acid development and causes urinary excretion to be neutralized by ammonia and phosphorus [65]. Meanwhile, the urine pH showed that *M. elsdenii* supplementation tended to decrease (p = 0.064), which also affected the interaction with livestock species and inoculation methods due to the similarity of the data obtained. Decrease in urine pH was associated with a decrease in DCAD, which was related to the fraction and grain size of the feed [36,65].

## CONCLUSION

The evaluation of *M. elsdenii* probiotic inoculation *in vivo* on fermentability, microbial population, performance, carcass quality, blood and urine metabolites from various articles indicates that it can increase several products of rumen fermentation, including total VFA, the proportion of propionate, butyrate, isobutyrate and valerate, while significantly decreasing lactic acid concentration, acetate proportion, bacterial population, and methane estimation. The increase in several rumen fermented products after supplementation with *M. elsdenii* correlated with increasing livestock performance, especially in the FBW, ADG, and BCS parameters, as well as an increase in the carcass quality parameters of hot carcass weight, carcass gain and dressing percentage. Livestock health after *M. elsdenii* supplementation also improved, in cattle with diarrhoea and bloat decreased, followed by a decrease in rumen score and liver score. However, probiotic supplementation had no effect on several blood and urine metabolites except for blood lactate and urine pH which tended to decrease. Since most of the parameters revealed linear trends by increasing levels of *M. elsdenii*, including some key parameters such as rumen pH, lactic acid concentration, ADG and DnB incidence, supplementation level of the probiotic up to 13.30 log 10 CFU was still appropriate for ruminants.

## Figures and Tables

**Table 1:** Studies used for meta-analysis of *Megasphaera elsdenii* supplementation in ruminants in vivo

Studies	Reference	Animal	Concentrate: forage ratio	Inoculation method	Strain	Level *M. elsdenii* (log 10 CFU)
1	Henning et al [13]	Sheep and cow	Up to 94:6	Canula rumen	CH4	0; 9.24; 10.24; and 11.24
2	Aikman et al [14]	Cow	61:39	Fistula rumen	NCIMB 41125	0 and 10.35
3	DeClerck et al [15]	Cow	Up to 90:10	Oral	NCIMB 41125	0 and 10.3
4	Miller et al [17]	Cow	Up to 90:10	Oral	NCIMB 41125	0 and 11
5	Ellerman et al [18]	Cow	Up to 90:10	Oral	NCIMB 41125	0 and 11.7
6	Henning et al [27]	Sheep	-	Canula rumen	CH4; CH7	0 and 11
7	Alatas and Umucalilar [28]	Sheep	60:40	Canula rumen	ATCC 17753	0 and 12.38
8	Hagg et al [29]	Cow	70:30	Oral	NCIMB 41125	0 and 11.40
9	Long et al [30]	Sheep	80:20	Fistula rumen	H6; H6F32	0 and 10.00
10	Sedighi and Alipour [31]	Cow	60:40	Oral	SA3	0 and 11.18
11	Muya et al [32]	Cow	-	Oral	NCIMB 41125	0 and 9.70
12	Weimer et al [33]	Cow	Up to 53:47	Canula rumen	YI-9; 4251; 4257; 4291	0; 12.26; 12.30; 12.34; and 12.41
13	Zebeli et al [34]	Cow	55:45	Canula rumen	ATCC 25940	0 and 9.54
14	Mazon et al [35]	Cow	Up to 71:29	Oral	NCIMB 41125	0 and 10.30
15	Arik et al [36]	Cow	Up to 80:20	Canula rumen	ATCC 17753	0 and 12.64
16	Direkvandi et al [37]	Sheep	70:30	Oral	GU1	0 and 8.65
17	Thieszen et al [38]	Cow	Up to 92:8	Oral	NCIMB 41125	0; 11.40; 11.70; and 11.88
18	McDaniel et al [39]	Cow	67:33	Canula rumen	NCIMB 41125	0; 9.21; 10.21; and 11.21
19	Leeuw et al [40]	Cow	Up to 80:20	Oral	NCIMB 41125	0 and 11.3
20	DeClerck et al [41]	Cow	Up to 90:10	Oral	NCIMB 41125	0 and 10.3
21	DeClerck et al [42]	Cow	Up to 90:10	Oral	NCIMB 41125	0 and 10
22	Drouillard et al [43]	Cow	Up to 94:6	Oral	NCIMB 41125	0 and 13.30
23	Muya et al [44]	Cow	-	Oral	NCIMB 41125	0 and 9.69
24	Klieve et al [45]	Cow	Up to 75:25	Canula rumen	YE34	0 and 11.74
25	Klieve et al [46]	Cow	Up to 89:11	Oral	YE34	0 and 11.12
26	Miller et al [47]	Cow	Up to 90:10	Oral	NCIMB 41125	0 and 11
27	Stevens et al [48]	Cow	Up to 60:40	Oral	NCIMB 41125	0 and 10.3
28	Veloso et al [49]	Cow	Up to 93:7	Oral	NCIMB 41125	0; 7 and 10
29	Direkvandi et al [50]	Sheep	70:30	Oral	GU1	0 and 8.65
30	Leeuw et al [51]	Cow	Up to 98:2	Oral	NCIMB 41125	0 and 11.3
31	Ye and Eastridge [52]	Cow	49:51	Oral	NCIMB 41125	0 and 10.3
32	Lopez et al [53]	Cow	75:25	Oral	NCIMB 41125	0 and 11.3

**Table 2:** Descriptive statistics on the effects of *Megasphaera elsdenii* supplementation levels on rumen fermentability, productivity, health, blood, and urine metabolite of ruminants *in vivo*

Response parameters	Unit	N	Min	Max	Mean	Std. Deviation
Level *M. elsdenii*	log10cfu	73	7.00	13.30	10.80	1.35
Fermentability						
pH		84	5.00	6.80	6.01	0.34
NH_3_	mM	26	5.05	24.80	11.01	4.53
Total VFA	mM	81	28.40	250.00	109.66	37.00
C_2_	%	76	32.53	78.90	53.28	9.60
C_3_	%	81	3.90	42.30	23.04	5.83
C_4_	%	76	1.12	40.40	14.62	6.45
Iso-C_4_	%	33	0.24	2.65	1.47	0.62
C_5_	%	43	0.20	8.71	3.03	1.83
Iso-C_5_	%	29	0.61	5.00	2.83	1.33
C_6_	%	10	0.00	0.93	0.41	0.26
Ratio C_2_:C_3_		76	1.16	8.45	2.62	1.13
Lactic acid	mmol/L	65	0.05	31.00	2.07	4.55
Bacteria	log10/mL	19	7.70	9.38	8.56	0.52
Protozoa	log10/mL	19	4.95	6.26	5.69	0.41
CH_4_	mM	76	13.34	34.40	23.58	3.84
Productivity						
DMI	kg/d BW^0.75^	124	1.11	13.33	6.53	2.77
IBW	kg BW^0.75^	124	10.94	145.46	78.03	40.06
FBW	kg BW^0.75^	54	15.09	120.85	87.09	32.81
ADG	kg BW^0.75^	56	0.30	2.24	1.46	0.45
FCR	kg/kg	36	2.50	6.63	4.60	0.91
BCS		16	2.55	4.80	3.38	0.76
Marbling		26	289.00	520.00	424.04	72.97
Conformation		18	3.10	3.31	3.22	0.07
Fatcode		18	2.15	2.30	2.21	0.04
LMA	cm^2^	10	66.03	101.40	82.66	12.77
KPH Fat	%	12	1.24	2.52	2.12	0.46
12th Rib Fat	cm^2^	24	0.38	1.78	0.68	0.32
HCW	kg BW^0.75^	44	52.58	90.28	70.96	11.15
Carcass gain	kg/d BW^0.75^	36	1.06	2.05	1.32	0.21
Dressing	%	40	50.50	63.80	58.61	3.61
Health						
Rumen score		18	0.45	0.80	0.62	0.09
Liver score		24	1.12	2.13	1.80	0.25
Liver abscess	%	16	4.70	2.10	12.81	5.35
DnB		18	3.00	19.00	7.78	4.53
Blood						
pH		15	7.00	7.43	7.38	0.10
PCO_2_	mmHg	15	39.74	44.79	42.57	1.55
Na	mM	13	146.00	150.00	147.23	1.09
K	mM	13	3.20	3.45	3.35	0.08
Ca	mM	13	0.72	0.86	0.80	0.05
Glucose	mg/dL	21	46.00	656.00	91.91	129.54
BHBA	mmol/L	14	0.17	18.60	5.91	7.08
Lactate	mM	17	0.38	0.87	0.57	0.15
Hematokrit	%	13	28.21	31.87	30.15	1.30
HCO_3_	mmol/L	13	24.00	28.10	26.63	1.17
BE	mmol/L	13	0.34	3.80	2.02	1.02
Urine						
pH		13	6.66	7.93	7.60	0.42
Na	mEq/L	13	66.00	174.00	109.88	33.53
K	mEq/L	13	122.00	248.00	193.08	44.48
Cl	mEq/L	13	87.00	207.00	141.23	37.88
EB	mEq/L	13	75.00	281.00	162.62	57.33

N, number of observations; NH_3_, ammonia; VFA, volatile fatty acid; C_2_, acetate; C_3_, propionate; C_4_, butyrate; Iso-C_4_, isobutyrate; C_5_, valerate; Iso-C_5_, isovalerate; C_6_, caproate; CH_4_, methane; ns, non-significant; DMI, dry matter intake; IBW, initial body weight; FBW, final body weight; ADG, average daily gain; BCS, body condition score; LMA, longissimus muscle area; KPH Fat, kidney pervic and heart fat; HCW, hot carcas weight; DnB, diarrhoea and bloat; BHBA, β-hidroxybutiric acid; BE, base excess; EB, electrolyte balance.

**Table 3:** Regression equations of rumen fermentation parameters in response to *Megasphaera elsdenii* supplementation

Response parameters	N	M	Parameter estimates				Model estimates			Interaction		IT
		
			Int	SE Int	Slope	SE slope	p-value	RMSE	AIC	Level vs animal	Level vs method	
pH	84	L	5.80	0.07	0.026	0.005	<0.001	0.543	45.8	0.470	0.885	+
NH_3_ (mM)	26	Q	11.43	1.28	2.154	1.037	0.049					
					−0.188	0.089	0.046	7.832	137.8	0.346	0.169	+
Total VFA (mM)	81	Q	99.26	5.14	−15.96	4.406	<0.001					
					1.481	0.377	<0.001	61.313	739.4	0.961	0.886	−
C_2_ (%)	76	L	57.67	2.05	−0.628	0.105	<0.001	17.316	504.8	0.419	0.551	−
C_3_ (%)	81	L	20.52	1.22	0.507	0.081	<0.001	10.299	491.9	0.604	0.642	+
C_4_ (%)	76	L	11.69	1.24	0.444	0.084	<0.001	10.686	463.4	0.116	0.933	+
Iso-C_4_ (%)	33	L	1.18	0.24	0.027	0.009	0.011	1.039	49.4	0.168	0.694	+
C_5_ (%)	43	L	2.10	0.51	0.096	0.049	0.058	3.58	189.2	0.428	0.154	ns
Iso-C_5_ (%)	29	L	2.08	0.49	−0.013	0.016	0.437	1.498	73.4	0.958	0.533	ns
C_6_ (%)	10	L	0.33	0.16	0.0004	0.008	0.959	0.591	13.1	0.959	0.118	ns
Ratio C_2_:C_3_	76	L	3.02	0.24	−0.077	0.020	<0.001	2.151	247.7	0.944	0.069	−
Lactic acid (mmol/L)	65	L	3.44	1.04	−0.195	0.086	0.028	8.131	385.5	0.459	0.047	−
Bacteria (log10/mL)	19	Q	8.43	0.12	0.278	0.105	0.019					
					−0.022	0.009	0.041	0.697	38.0	0.059	0.056	+
Protozoa (log 10/mL)	19	L	5.73	0.26	0.01	0.014	0.512	0.548	25.9	0.379	0.083	ns
CH_4_ (mM)	76	L	24.96	0.84	−0.235	0.057	<0.001	7.158	405.8	0.970	0.668	−

n, number of observations; M, model; L, linear; Q, quadratic; Int, intercept; SE, standard error; RMSE, root mean square error; AIC, Akaike information criterion; IT, interpretation trend; NH_3_, amonnia; VFA, volatile fatty acid; C_2_, acetate; C_3_, propionate; C_4_, butyrate; Iso-C_4_, isobutyrate; C_5_, valerate; Iso-C_5_, isovalerate; C_6_, caproate; CH_4_, methane; ns, non-significant; +, increasing trend; −, decreasing trend.

**Table 4:** Regression equations of performance and carcass parameters in response to *Megasphaera elsdenii* supplementation

Response parameters	n	M	Parameter estimates				Model estimates			Interaction		IT
		
			Int	SE Int	Slope	SE slope	p-value	RMSE	AIC	Level vs Animal	Level vs Method	
DMI (kg/d BW^0.75^)	124	L	12.27	1.07	−0.089	0.027	0.001	13.142	592.0	0.462	0.783	−
IBW (kg BW^0.75^)	124	L	69.86	7.72	0.032	0.085	0.710	62.392	619.9	0.843	0.485	ns
FBW (kg BW^0.75^)	54	L	79.27	10.02	0.193	0.065	0.005	46.64	356.7	0.718	0.691	+
ADG (kg BW^0.75^)	56	L	1.27	0.13	0.007	0.003	0.02	0.743	−9.8	<0.001	0.823	+
FCR	36	L	4.52	0.38	−0.008	0.011	0.482	1.502	61.2	0.096	0.314	ns
BCS	16	L	3.39	0.33	0.03	0.011	0.026	1.433	29.1	0.587	0.587	+
Marbling	26	L	424.22	25.19	0.351	0.432	0.429	164.48	237	0.892	0.892	ns
Conformation	18	L	3.24	0.02	−0.003	0.003	0.37	0.051	−28.4	0.369	0.369	ns
Fat Code	18	L	2.21	0.01	−0.008	0.002	0.668	0.071	−47	0.668	0.668	ns
LMA (cm^2^)	10	L	81.82	7.72	−0.01	0.057	0.864	30.109	55.6	0.911	0.911	ns
KPH Fat (%)	12	Q	2.10	0.17	−0.767	0.323	0.042					
					0.073	0.029	0.036	0.623	8.9	0.224	0.224	−
12th Rib Fat (cm^2^)	24	L	1.02	0.12	−0.041	0.011	0.002	0.843	32.2	0.001	0.001	−
HCW (kg BW^0.75^)	44	L	72.39	3.56	0.045	0.016	0.012	29.45	160.9	0.274	0.274	+
Carcas Gain (kg/d BW ^0.75^)	36	L	1.38	0.1	0.004	0.001	0.048	0.353	−57	0.274	0.274	+
Dressing (%)	40	L	58.06	1.32	0.09	0.045	0.062	6.932	184.2	0.029	0.029	ns

n, number of observations; M, model; L, linear; Q, quadratic; Int, intercept; SE, standard error; RMSE, root mean square error; AIC, Akaike information criterion; IT, interpretation trend; DMI, dry matter intake; IBW, initial body weight; FBW, final body weight; ADG, average daily gain; BCS, body condition score; LMA, longissimus muscle area; KPH fat, kidney pervic and heart fat; HCW, hot carcas weight; ns, non-significant; +, increasing trend; −, decreasing trend.

**Table 5:** Regression equations of health parameters in response to *Megasphaera elsdenii* supplementation

Response parameters	n	M	Parameter estimates				Model estimates			Interaction		IT
		
			Int	SE Int	Slope	SE slope	p-value	RMSE	AIC	Level vs Animal	Level vs Method	
Rumen score	18	L	0.59	0.03	0.006	0.003	0.101	0.134	−24.7	0.129	0.129	ns
Liver score	24	L	1.7	0.13	−0.003	0.007	0.715	0.394	4.4	0.735	0.735	ns
Liver abscess (%)	16	L	16.64	2.34	−0.405	0.163	0.032	12.855	94.1	0.037	0.037	-
DnB	18	L	10.76	1.64	−0.573	0.148	0.001	5.774	95.3	0.002	0.002	-

n, number of observations; M, model; L, linear; Q, quadratic; Int, intercept; SE, standard error; RMSE, root mean square error; AIC, Akaike information criterion; IT, interpretation trend; DnB, diarrhoea and bloat; ns, non-significant; -, decreasing trend.

**Table 6:** Regression equations of blood metabolite parameters in response to *Megasphaera elsdenii* supplementation

Response parameters	N	M	Parameter estimates				Model estimates			Interaction		IT
		
			Int	SE Int	Slope	SE slope	p-value	RMSE	AIC	Level vs Animal	Level vs Method	
pH	15	L	7.41	0.01	0.0009	0.0007	0.241	0.021	−61.1	0.161	0.959	ns
PCO_2_ (mmHg)	15	L	42.56	0.53	−0.026	0.086	0.765	2.348	62.3	0.748	0.469	ns
Na (mM)	13	L	147.27	0.34	−0.022	0.069	0.761	1.606	44.2	0.761	0.761	ns
K (mM)	13	L	3.35	0.02	−0.004	0.004	0.451	0.112	−14.4	0.451	0.451	ns
Ca (mM)	13	L	0.79	0.01	0.003	0.002	0.289	0.065	−26.5	0.289	0.289	ns
Glucose (mg/dL)	21	L	73.58	37.71	6.744	5.384	0.228	183.14	250	0.639	0.386	ns
BHBA (mmol/L)	14	L	6.34	3.7	0.049	0.029	0.138	10.099	57.1	0.731	0.969	ns
Lactate (mM)	17	L	0.68	0.13	−0.011	0.005	0.054	0.242	−10	0.487	0.487	ns
Hematokrit (%)	13	L	30.34	0.38	−0.094	0.077	0.253	1.808	46.8	0.253	0.253	ns
HCO_3_ (mmol/L)	13	L	26.52	0.36	0.057	0.072	0.448	1.677	45.1	0.448	0.448	ns
BE (mmol/L)	13	L	1.92	0.34	0.053	0.066	0.442	1.535	40.1	0.442	0.442	ns

n, number of observations; M, model; L, linear; Q, quadratic; Int, intercept; SE, standard error; RMSE, root mean square error; AIC, Akaike information criterion; IT, interpretation trend; BHBA, beta-hidroxybutiric acid; BE, base excess; ns, non-significant.

**Table 7:** Regression equations of urine electrolyte parameters in response to *Megasphaera elsdenii* supplementation

Response parameters	n	M	Parameter estimates				Model estimates			Interaction		IT
		
			Int	SE Int	Slope	SE slope	p-value	RMSE	AIC	Level vs Animal	Level vs Method	
pH Urine	13	L	7.68	0.11	−0.046	0.022	0.064	0.523	19.5	0.064	0.064	ns
Na (mEq/L)	13	L	114.41	9.97	−2.326	2.011	0.272	46.761	118.3	0.272	0.272	ns
K (mEq/L)	13	L	195.37	13.89	−1.176	2.803	0.683	65.186	125.7	0.683	0.683	ns
Cl (mEq/L)	13	L	141.82	11.92	−0.302	2.404	0.902	55.911	122.3	0.902	0.902	ns
EB (mEq/L)	13	L	169	17.37	−3.283	3.504	0.369	81.499	130.6	0.369	0.369	ns

n, number of observations; M, model; L, linear; Q, quadratic; Int, intercept; SE, standard error; RMSE, root mean square error AIC, akaike information criterion; IT, interpretation trend; EB, electrolyte balance; ns, non-significant.
